# Rare observations of sprites and gravity waves supporting D, E, F-regions ionospheric coupling

**DOI:** 10.1038/s41598-021-03808-5

**Published:** 2022-01-12

**Authors:** Ajeet K. Maurya, Navin Parihar, Adarsh Dube, Rajesh Singh, Sushil Kumar, Olivier Chanrion, Maja Tomicic, Torsten Neubert

**Affiliations:** 1 Department of Physics, Babasaheb Bhimrao Ambdekar University, Lucknow, India; 2 Indian Institute of Geomagnetism, Navi Mumbai, Tirunelveli, India; 3 KSK Geomagnetic Research Laboratory, IIG, Prayagraj, Allahabad, India; 4 School of Information Technology, Engineering, Mathematics and Physics, The University of South Pacific, Suva, Fiji; 5 National Space Institute, Technical University of Denmark (DTU Space), Kongens Lyngby, Denmark; 6 Present Address: Indian Institute of Tropical Meterology, MoES, Pune, India

**Keywords:** Atmospheric science, Climate sciences, Space physics

## Abstract

We report rare simultaneous observations of columniform sprites and associated gravity waves (GWs) using the Transient Luminous Events (TLEs) camera and All-sky imager at Prayagraj (25.5° N, 81.9° E, geomag. lat. ~ 16.5° N), India. On 30 May 2014, a Mesoscale Convective System generated a group of sprites over the north horizon that reached the upper mesosphere. Just before this event, GWs (period ~ 14 min) were seen in OH broadband airglow (emission peak ~ 87 km) imaging that propagated in the direction of the sprite occurrence and dissipated in the background atmosphere thereby generating turbulence. About 9–14 min after the sprite event, another set of GWs (period ~ 11 min) was observed in OH imaging that arrived from the direction of the TLEs. At this site, we also record Very Low Frequency navigational transmitter signal JJI (22.2 kHz) from Japan. The amplitude of the JJI signal showed the presence of GWs with ~ 12.2 min periodicities and ~ 18 min period. The GWs of similar features were observed in the ionospheric Total Electron Content variations recorded at a nearby GPS site. The results presented here are important to understand the physical coupling of the troposphere with the lower and upper ionosphere through GWs.

## Introduction

Gravity waves (GWs) are the important dynamical drivers of the coupling of atmosphere–ionosphere system. Being involved in the energy and momentum transfer processes, GWs play a critical role in understanding atmospheric coupling. Numerous studies have reported GWs associated with thunderstorms/lightning discharges, however, their generation mechanism is still not well understood^1–6^. Stull^1^ suggested that the convection in a thunderstorm could effectively launch upward propagating GWs with a period near the Brunt-Vaisala period at the source altitude. Pasko et al.^2^ reported that the Transient Luminous Events (TLEs) like Sprites can be associated with the large mesoscale thunderstorm generated GWs which modulate atmospheric density at mesospheric altitudes. Sentman et al.^3^ proposed that localized Joule heating within the sprite volume could be the source of GWs since TLEs are short-duration (lasting for a few milliseconds) phenomena associated with the electromagnetic pulse (EMP) of strong thunderstorms. Hence, thermal energy deposited in the neutral atmosphere within a sprite is expected to produce an impulsive pressure pulse that propagates laterally outward and acts as a source of acoustic waves or GWs. However, they were not able to observe any impulsive pressure pulse. They have further denied such possibility of a sprite effect on OH emission through thermal heating due to the large energy difference between the energy deposited by sprite (1–10 MJ) and energy required (~ 1 GJ) to produce a detectable response in OH emission. Therefore, the interconnection between sprites and GWs remained not well understood. This is probably because limited reports exist in the literature on the simultaneous observations of sprites and GWs, and their interconnections^4,7^. Sentman et al.^3^ presented simultaneous imaging under the NASA Sprites99 balloon campaign; and Siefring et al.^7^ reported simultaneous observations under the Energetics of Upper Atmospheric Excitation by Lightning 1998 (EXL98) airborne campaign. Both the studies featured sprite associated GWs in OH broadband airglow imaging. Siefring et al.^7^ found that a distance of about ~ 400–800 km is required from the sprite location to observe GWs correlated with sprites. Barta et al.^8^ studied two cases of simultaneous observations of TLEs and sporadic E (Es) layer over two stations in Central Europe and found a reduction and disappearance of Es layer during the thunderstorm activity. They have suggested that thunderstorm generated GWs can propagate to the E-region and may cause reduction and even disappearance of the Es layer. The majority of these investigations featured the sprites associated with GWs at the mesosphere-lower thermosphere (MLT) heights. Except Barta et al.^8^, these reports did not discuss the response of ionospheric D, E, F regions to GWs.

Numerical simulations by *Snively and Pasko*^4^ indicated that thunderstorms can generate GWs and that these GWs upon reaching the MLT heights can break and excite secondary GWs. Using corroborative study involving Next Generation Weather Radar (NEXRAD) thunderstorm measurements and the ionospheric acoustic and GWs detected in Global Positioning System (GPS) based Total Electron Content (TEC) measurements, Lay et al.^6^ found that the areas disturbed by acoustic waves (AWs) and GWs increased with the enhancement in the thunderstorm activity. Although these observations suggested that GWs from thunderstorm/convection can reach to the lower ionosphere and upper ionosphere but their simultaneous observations at a given location are still lacking. Recently, Maurya et al.^9^ reported the simultaneous presence of thunderstorm/convection generated GWs in form of wave-like fluctuations in the VLF signal amplitude and the GPS TEC in the same region, which provided probably the first simultaneous observations of GWs in the lower and upper ionosphere but did not observe any associated TLE (sprite) event.

In this study, we present rare simultaneous imaging observations of sprites and GWs using a TLE camera and an all-sky airglow imager. We also investigate the GWs and associated physical coupling of the D-, E- and F- regions during a Mesoscale Convective System (MCS) event using airglow, VLF and TEC measurements.

## Observations

### MCS Meteorological conditions and sprite observations

**Figure 1:**
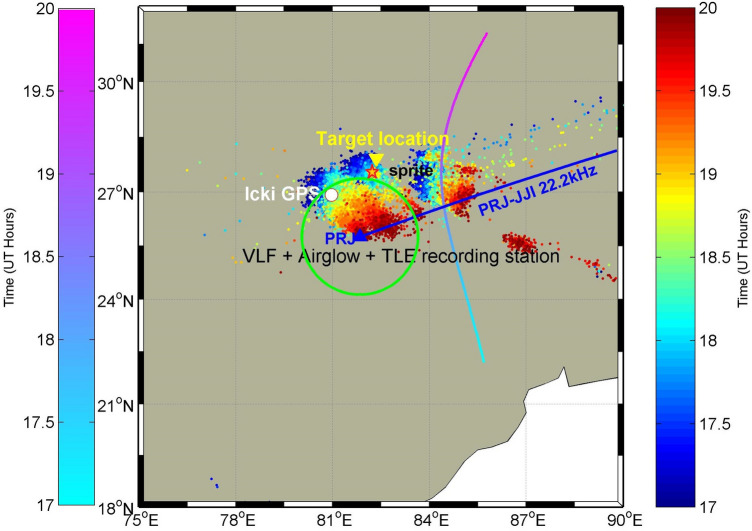
Map depicts the geographic location of Prayagraj (PRJ) (formerly Allahabad), India, where VLF experiment, Airglow imager, and TLE camera are located. The green circle represents the field-of-view of the airglow imager at the MLT heights. IGS GPS receiver at Lucknow (LCKI) is shown by the small white circle. We aimed the center of the camera field-of-view at the target TLE location indicated by the downward yellow triangle. The estimated sprite location is marked by a yellow–red color star. The JJI (22.2 kHz) VLF signal path to PRJ is shown with the blue line, whereas the satellite path for PRN 26 and lightning activity from GLD360 are depicted color-coded with time in UT during 17–20 UT.

**Figure 2:**
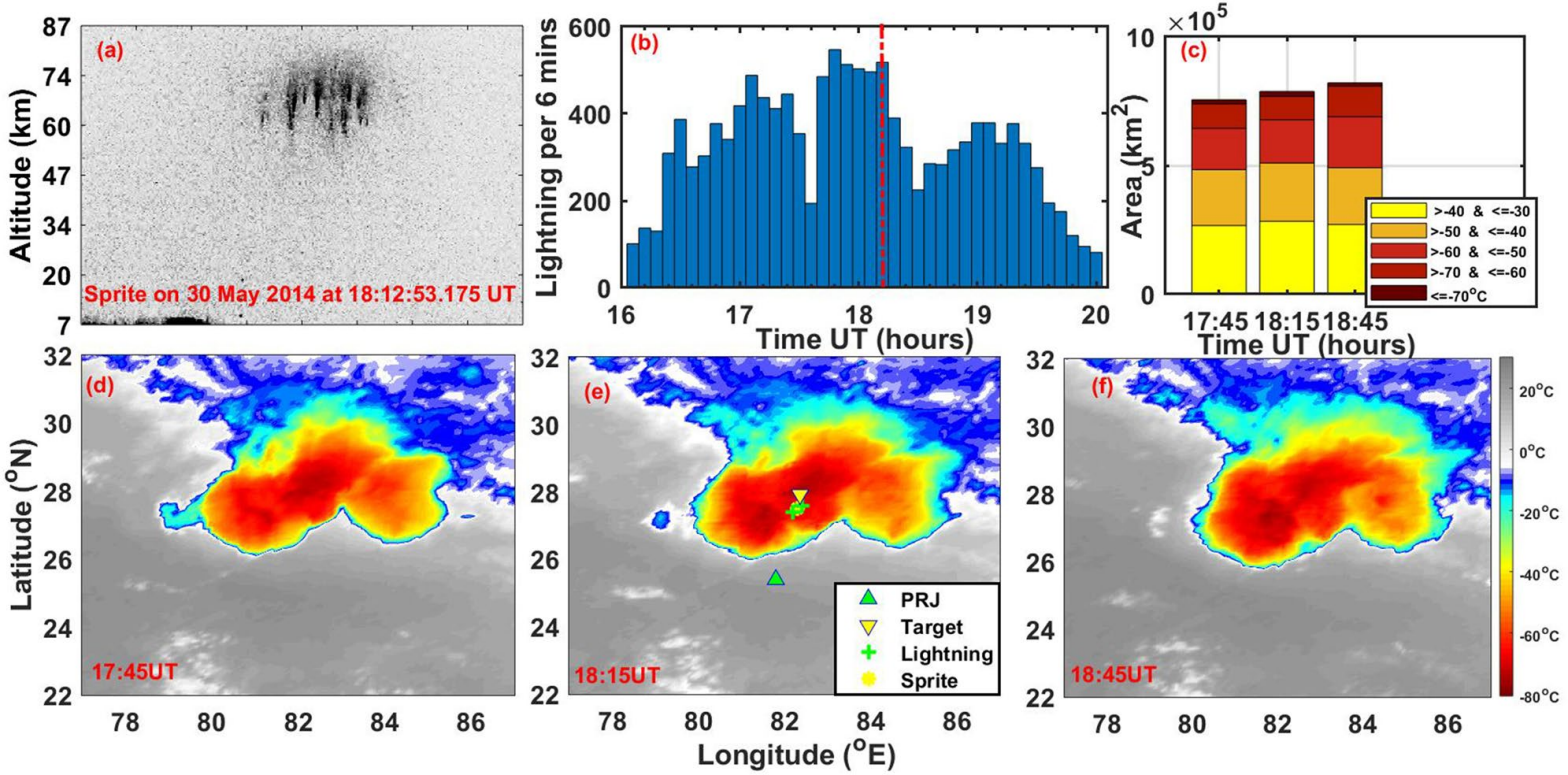
(**a**) Sprite event recorded on 30 May 2014 at 18:12:53.175 UT. The altitude scale corresponds to a distance of camera-sprite event of 260 km. (**b**) The lightning occurrence rate per 6 min for + CG and − CG lightning during (16–20 UT) was obtained from the GLD360 lightning location network. (**c**) The cloud top area variation for different temperature ranges at 17:45, 18:15 and 18:45 UT. (**d**–**f**) presents cloud top temperature at 17:45, 18:15 and 18:45 UT obtained from Kalpana1 meteorological satellite IR brightness temperature data. We aimed the center of the camera field-of-view at a target TLE location indicated by the downward yellow triangle. The two green overlapping crosses indicate the locations of the lightning used to estimate the sprite location which is given by a yellow star.

On 30 May 2014, an intense MCS event occurred over the region under investigation during the period 16–24 UT. Lightning discharges associated with the MCS event are shown in Fig. 1. During this period, a few hours of clear sky occurred over the Prayagraj station which provided an opportunity for the operation of the TLE camera and Airglow experiments. Figure 2a shows a sprite event recorded at 18:12:53 UT. The TLE imaging system was pointing towards the north at an azimuth of 8.5 deg with an elevation of about 10 deg, observing a thunderstorm located ~ 270 km north–east of Prayagraj. The thunderstorm location is obtained by looking at the satellite image. We observed a group of sprites fully developed all in one frame (wide-angle camera) with 20 ms exposure time. The sprite event occurred between 18:12:53.155 and 18:12:53.175 UT. As the sprites were observed only in one frame, we presume that they had a lifetime within 20 ms in agreement with the sprite duration observed globally which varies in the range of a few ms to ~ 100 s of ms^10,11^. The approximate sprite altitude is estimated between ~ 55 km and 80 km in agreement with the range of sprite altitude reported normally^12,13^. It is to be noted that at this distance, the field-of-view of the camera was vertical and horizontal with dimensions of 78 km × 94 km centered at an altitude of 47 km. Despite this large coverage, only one group of sprites was observed. Though not very clearly seen most of the sprites appeared as vertical columns, hence they may be classified as columniform sprites^14^.

Figure 2b shows the lightning activity per 6 min recorded by the GLD360 lightning location network within 240 min duration starting from 16 UT. The occurrence time of the sprites event ~ 18:12:53 UT is indicated by a vertical broken red line. The occurrence rate for lightning activity shows a peak during ~ 17:40 UT–18:20 UT, which includes sprite occurrence timing as well. The high lightning occurrence peak suggests a presence of strong convection. No obvious candidates for the sprite parent lightning were detected by GLD360 network. Therefore, the sprite location is estimated by finding the distance from the camera that gives a sprite top altitude which matches its typical altitude of 80 km. It is also consistent with the fact that sprites usually occur above the stratiform region trailing the convective region (Lyons et al.^15^). Given the camera elevation and azimuth angle of 10° and 8.5°, respectively, the distance corresponding to a sprite top at 80 km altitude comes out 260 km resulting in a location of geog. lat. 27.73° N and geog. Long. 82.33° E which is indicated in Fig. 2e. The cloud top area evolution at 17:45 UT, 18:15 UT and 18:45 UT is shown in Fig. 2c. We have used *Kalpana 1* (Indian meteorological satellite) infrared (IR) brightness temperature data which are available every half an hour from the ISRO MOSDAC website (https://mosdac.gov.in/). The area is estimated for the different temperature ranges of 10 °C each starting from − 30 °C upto <  =  − 70 °C and shown for comparison as bar plots at 17:45 UT, 18:15 UT and 18:45 UT. The comparisons at three timings show that the area under the coldest temperature (< − 70 °C) was maximum at the sprite occurrence time (18:15 UT) (~ 19,812 km^2^) compared to at 17:45 UT (~ 16,738 km^2^) and 18:45 UT (~ 12,812 km^2^). Further, the area with temperature range of − 30 to − 40 °C and − 40 to − 50 °C was also highest at this time (18:15 UT), while the area with temperature range of − 50 to − 60 °C)and − 60 to  − 70 °C was highest at 18:45 UT. The cloud top temperature and area evolution suggest that the deep MCS was prevalent during the three analysis time considered here, but it was at its peak condition during sprite occurrence time (~ 18:12:53 UT). Further, it also appears that after 18:15 UT, the temperature started increasing and the area with higher temperature is larger than at 18:15 UT. Details of cloud top temperature at 17:45 UT, 18:15 UT and 18:45 UT are shown in Fig. 2d–f. The minimum temperature observed was − 73.3 °C, − 75.3 °C and − 73.6 °C at 17:45 UT, 18:15 UT and 18:45 UT, respectively. This indicates that the cloud top temperature was minimum close to sprite occurrence time.

### Observations of GWs in OH broadband nightglow imaging

**Figure 3:**
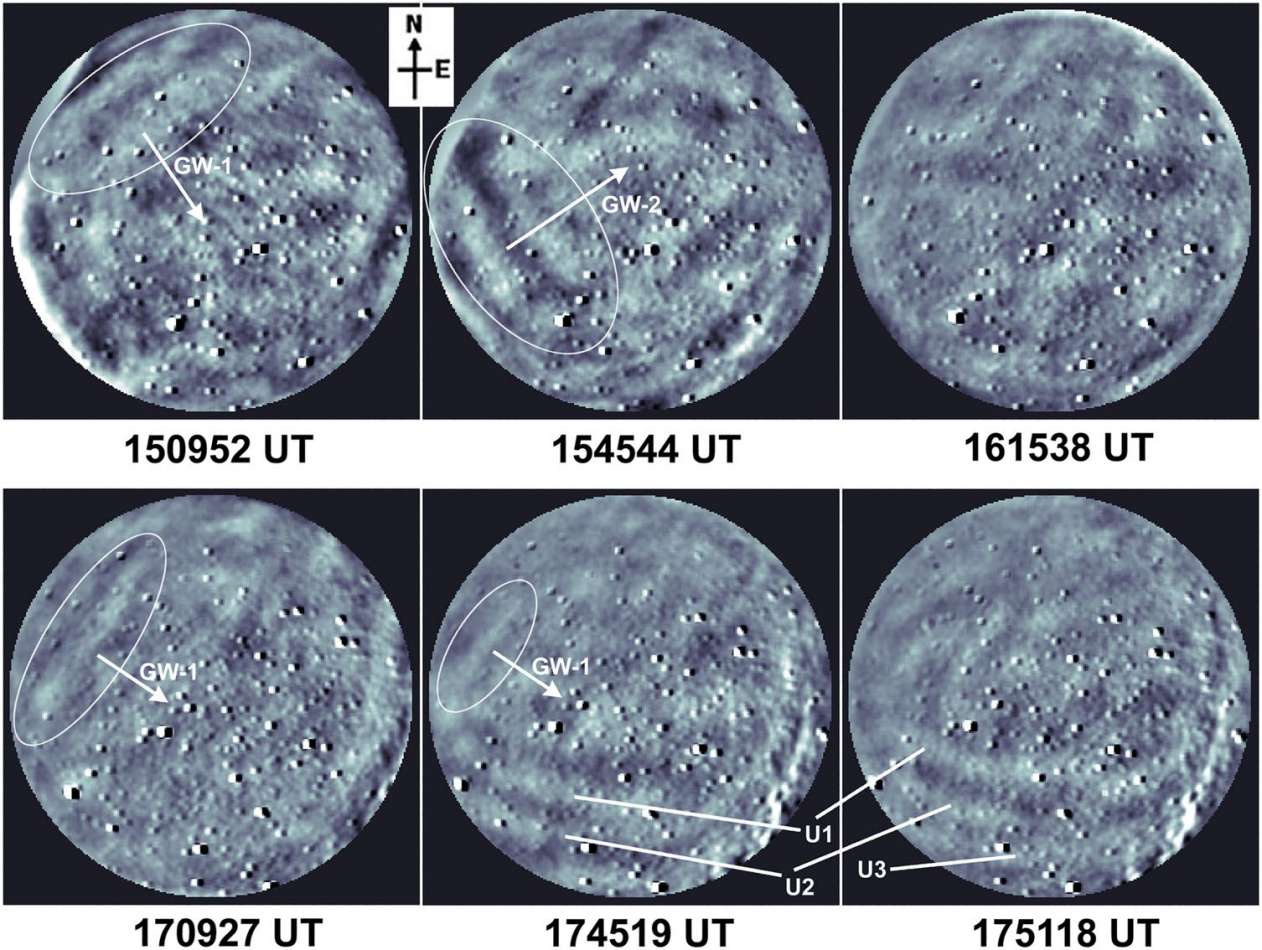
Sample TD OH broadband nightglow images during 15:00–18:00 UT featuring small-scale GWs seen over Prayagraj. GWs propagating towards the south-east and north-east are represented by GW-1 and GW-2, respectively. Some detected fronts of GWs that possibly modified the ionization rate and created suitable conditions for sprite initiation are marked by U1, U2, and U3.

**Figure 4:**
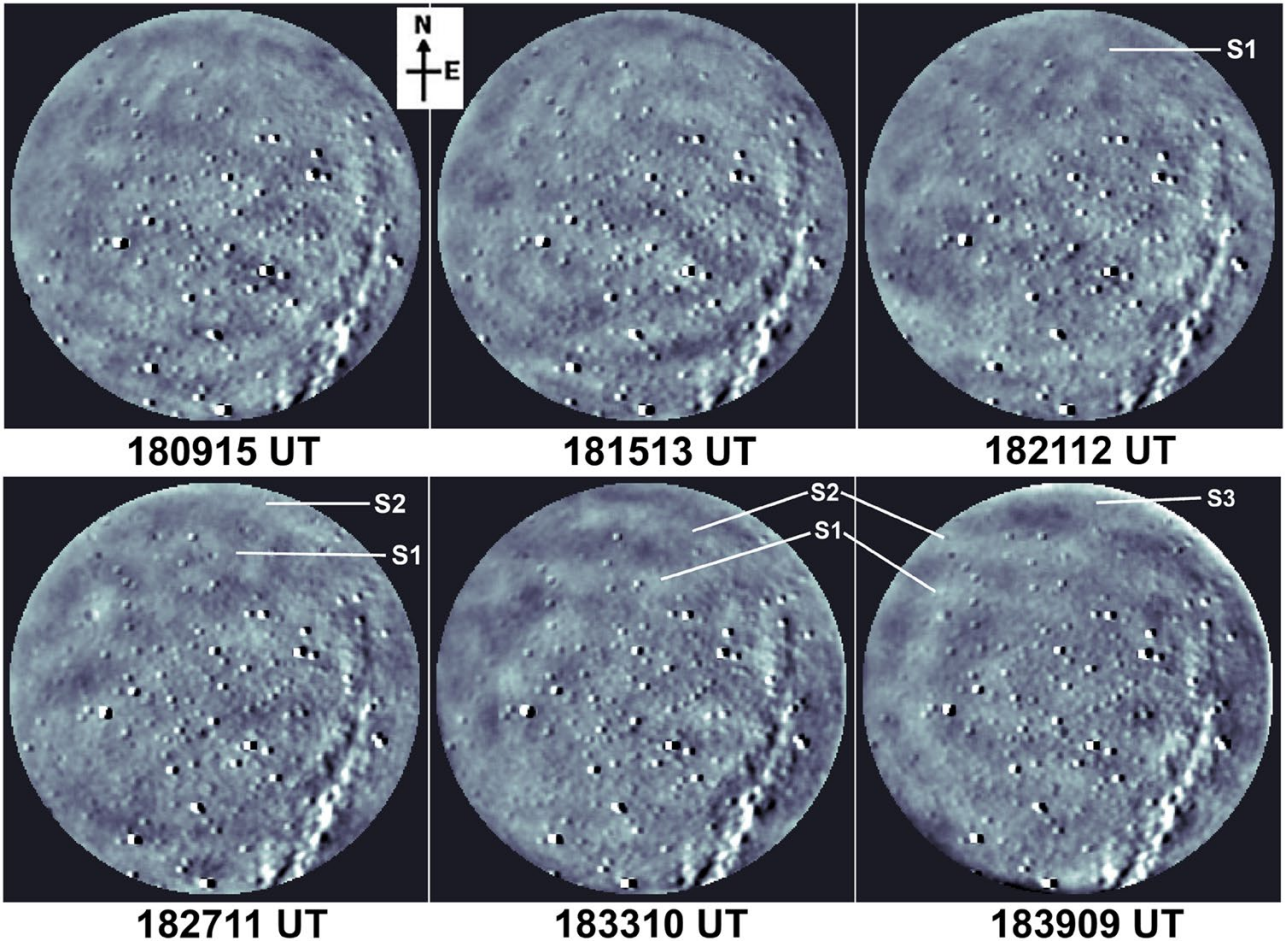
Same as Fig. 3 but for the interval 18:00–19:00 UT. GWs wavefronts arriving from the sprite occurrence location are indicated by S1, S2 and S3.

Figures 3 and 4 present OH broadband (705–929 nm) nightglow images recorded during 15:00–18:00 UT (before the TLE event) and 18:00–19:00 UT (around and after the TLE event at ~ 18:13 UT). At the beginning of imaging observations at ~ 15:00 UT, strong GW activity was noted at OH heights (87 ± 4 km). Since GWs signatures were weakly perceivable in raw OH broadband images, we generated time differenced (TD) images to bring out clearly the observed GWs^16^. Supplementary Movie (S1) presents a movie created from these TD OH broadband images during 14:52–19:03 UT. We observed two classes of GWs that propagated towards the south-east and towards the north-east (represented as GW-1 and GW-2, respectively, in Fig. 3). Unlike the OH layer, the OI 557.7 nm emission layer (97 ± 3 km) was fairly quiet in terms of GW activity. North–east propagating GWs are common over Prayagraj during the summer months. Using airglow imaging, Mukherjee et al.^17^ studied GWs motions over Allahabad (now Prayagraj) and found distinctly dominant waves propagating towards the northeast during April–May months. Hence, we assume these GW-2 waves to be usual GWs. Often the GW-1 and GW-2 wavefronts crossed each other (nearly orthogonally) thereby leading to the formation of distorted and unclear wave structures and a highly perturbed OH emission layer. Starting around 17:33 UT, we noted a set of GW-2 wavefronts over the southern horizon that propagated towards the north–east (horizontal wavelength ~ 33 ± 3 km, phase speed ~ 38 ± 6 m/s, and period ~ 14 min). Some distinct wavefronts are marked as U1, U2 and U3 in OH images during ~ 17:45–17:51 UT in Fig. 3. Similar wavefronts that almost reached the northern edge of the field-of-view were seen in OI 557.7 nm images as well during ~ 17:48–17:54 UT (shown in supplementary Fig. S2). Concurrent GW-1 wave activity was also noted at OH heights.

As these GW-2 wavefronts traversed the field-of-view during 17:57–18:09 UT, they interspersed with GW-1 structures and distorted wavefronts were seen which subsequently started disappearing, and turbulent regions were noted in the OH images. Around this time i.e. during ~ 18:00–18:12 UT, GW activity was not detected in 557.7 nm imaging which indicates the wave dissipation at OH heights. The sprites were seen in about 3 min over the north-horizon (outside the field-of-view of the imager) and afterwards a set of quasi-east–west aligned fronts (marked as S1, S2 and S3 in Fig. 4) were seen propagating towards the south-west and appeared to arrive from the direction of the location of MCS and sprites. Such quasi-east–west aligned and south-west propagating waves were first identified at ~ 18:21–18:27 UT and were seen until 18:45 UT. Unwarped (i.e. geographically corrected) images indicate that their fronts were planar. TD OI 557.7 nm nightglow images (having the emission peak at ~ 97 km) also showed weak signatures of these GWs which due to lack of clarity have not been presented here. We estimated the horizontal wavelength (*λ*_*H*_), phase speed (*υ*), period (*τ*) and azimuth (*φ*) of this set of GWs as ~ 60 ± 3 km, 89 ± 5 m/s, 11 min and 14 ± 1° azimuth (almost in line with the MCS and sprite occurrence direction), respectively. Airglow observations clearly showed strong GWs activity including wave dissipation around 87 km during 17:45–18:09 UT (i.e., in the half-an-hour period before the occurrence of sprite) and then GWs arriving from the sprite’s occurrence location during 18:21–18:45 UT. The possible source of these waves has been discussed in “Discussion” section.

### Determination of GWs from VLF signal amplitude data

**Figure 5:**
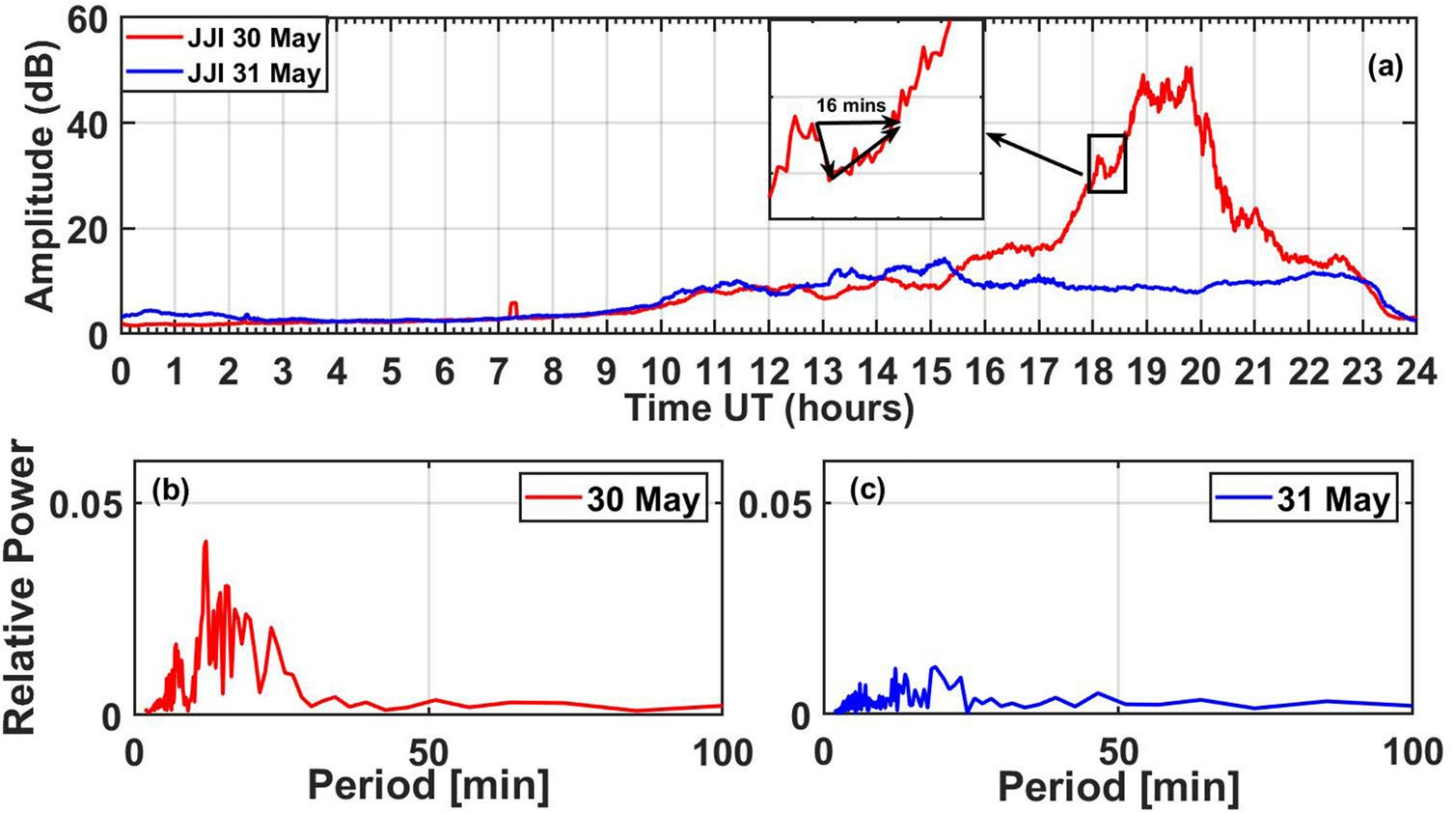
(**a**) JJI (22.2 kHz) VLF signal amplitude variation on 30 and 31 May 2014 with red and blue colors, respectively. The inset shows the zoomed of signal drop-in and recovery at the sprite occurrence time. (**b**) FFT of the JJI signal filtered amplitude during 17–20 UT on 30 May 2014. The highest peak shows dominant frequency ~ 12.2 min. (**c**) FFT of the JJI signal filtered amplitude during the same period on 31 May 2014.

Figure 5a presents the amplitude variation of JJI VLF transmitter signal (22.2 kHz) recorded at Prayagraj on 30 May (MCS days) and 31 May (Non-MCS day). The 31 May is chosen as a normal or unperturbed day for the comparison, based on the low lightning and geomagnetic activity and is close to the event day. The JJI signal on event day (30 May 2014) was very much enhanced during ~ 17–21 UT when compared with the signal on a normal day (31 May 2014) and this time. In Fig. 5a (inset) we also show a sudden decrease in the VLF signal amplitude on 30 May corresponding to the time of sprite occurrence i.e. 18:12:53 UT. The zoomed portion during 18:00–18:40 UT shows a sudden decrease in the signal amplitude corresponding to the time of sprite occurrence (black vertical line) which recovered after ~ 16 min. Since the sudden signal decrease took relatively a longer time (~ 1 min), it does not correspond to any known category (e.g., early/fast/slow/LORE etc.) reported in the previous works^18–21^. Further, after the decrease in JJI signal amplitude, it shows more fluctuations that lasted up to 21 UT, indicating a possible presence of GWs.

To look for the presence of any waves during the enhanced JJI signal, we have used the Fast Fourier Transform (FFT) technique to bring out the spectral features of the VLF signal to determine the signatures of GWs. Figure 5b shows the FFT analysis of JJI signal filtered amplitude during 17–20 UT, which exhibits a dominant period of ~ 12.2 min. We have used 5–20 min filtering range to keep short period GWs of interest and to filter long period as well as very short period waves. Generally, the waves of interest get masked under high period waves due to cyclic amplitude changes^22^. For the comparison purpose, FFT of the JJI filtered (5–20 min) amplitude for the same duration (17–20 UT) on 31 May 2014 is shown in Fig. 5c. The relative power of the FFT on 31 May shows a very small value as compared to FFT on 30 May, which indicates that there was no strong wave activity on 31 May.

### Observations of GWs inGPS TEC data

To see if GWs observed in the lower ionosphere propagated to the upper F-region ionosphere during the MCS on 30 May 2014, we have used GPS derived total electron content (TEC) data from IGS stations of Lucknow (LCKI) (located in MCS region). The carrier phase and code pseudo range measurements at different frequencies in various navigational systems are used to estimate the TEC, which is extensively used to study variation in the ionosphere^23^. Each satellite of the GPS is assigned a unique Pseudo Range Number (PRN), which allows any receiver to identify exactly which satellite(s) it is receiving the signal from. In this work, we have used PRN26 (shown in Fig. 1) based on the path coverage of the MCS observed during the period ~ 17–20 UT. An elevation mask > 30° is used to minimize the ionospheric effects during Vertical TEC (VTEC) data estimation. For comparison/reference GPS TEC data on 31 May 2014 for the same PRN26 is analyzed. As discussed by Lay et al.^24^, we first estimated differential VTEC (dVTEC) also known as the residue of VTEC by taking the difference of VTEC data with the best polynomial fit. The best polynomial fit is chosen with a minimized chi-square difference and the best accuracy of the fit. Thus 7th order polynomial was found suitable for event day (30 May) whereas the 9th order polynomial for the normal day (31 May).

**Figure 6:**
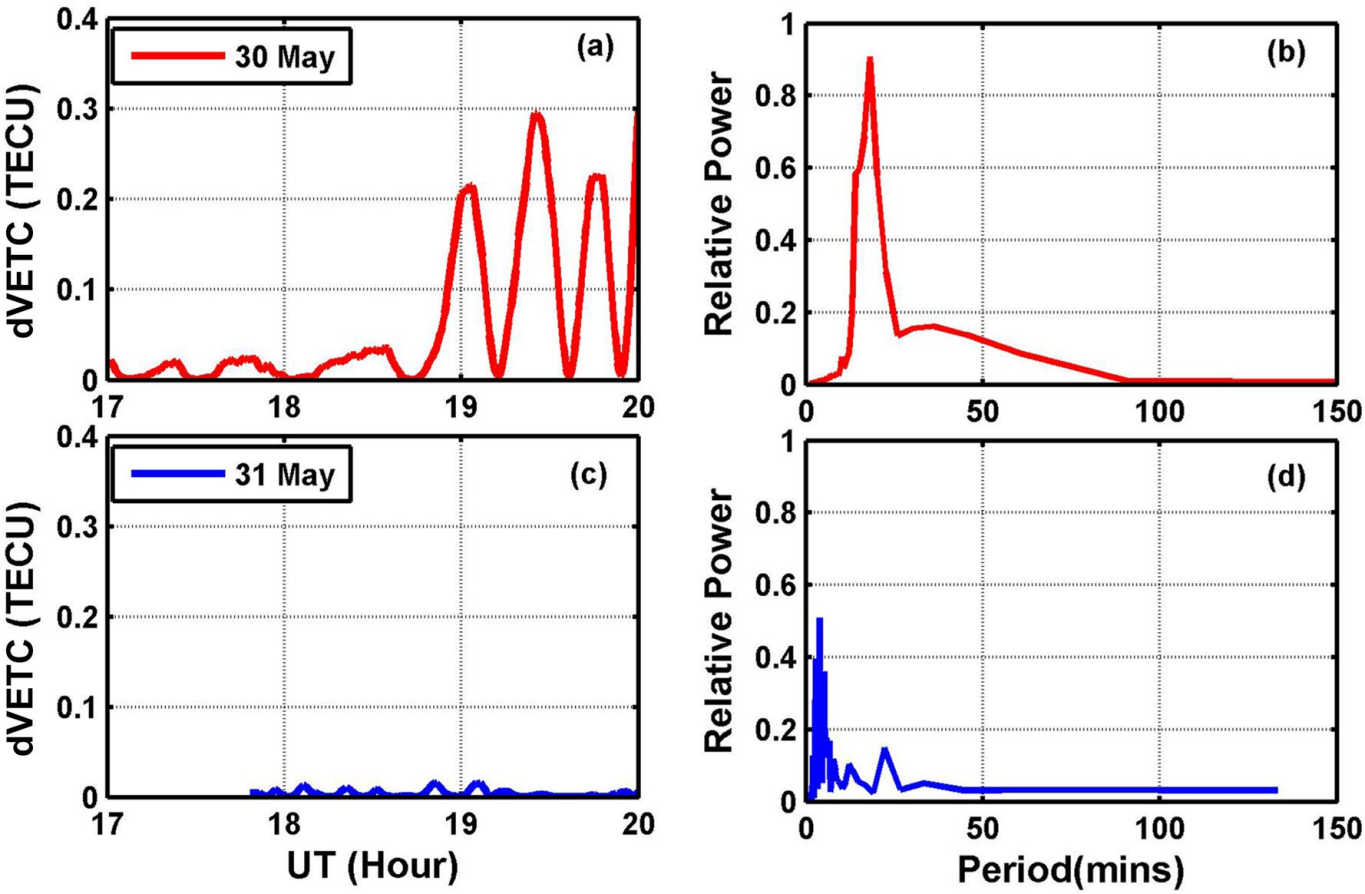
(**a**,**c**) dVTEC variation for Lucknow (LCKI) on 30 May and 31 May respectively (**b**,**d**) FFT of dVTEC at Lucknow on 30 May and 31 May 2014, respectively.

The filtered residue (dVTEC) variation for the Lucknow (LCKI) station is presented in Fig. 6a, c for 30 May and 31 May 2014, respectively. For the 30 May (Fig. 6a), the day of the event, it can be noted that dVTEC amplitude fluctuated from ~ 18:50 UT and increased slowly with time peaking at about 19:20 UT. For 31 May, a quiet day, the fluctuations in the dVTEC amplitude were very small (~ 0) as compared to on the event day. The enhancement in GPS-TEC over the IGS stations due to tropospheric convection is also reported in the recent work of Dube et al.^25^. We consider that the fluctuations in the dVTEC are associated with GWs observed in the OH airglow at ~ 18:21 UT and propagated upward to the F-region altitude (~ 250 km) with ~ 30 min time lag. The dVTEC data further analyzed by using the FFT technique for both days is presented in Fig. 6b, d. The FFT on event day (30 May) (Fig. 6b) shows a maximum peak of ~ 18 min at relative power of 0.9 (arbitrary unit). The FFT on a normal day (31 May) (Fig. 6d) shows much smaller relative power indicating quiet conditions.

## Discussion

We present simultaneous observations of an MCS event, a transient luminous event (sprite) at the MLT region, and the GW associated perturbations induced in the lower and upper ionosphere (D-, E- and F-region). Both sprites and GWs are generated by the thunderstorm; however, their simultaneous observations remain rare. To the authors' best knowledge, such simultaneous observations have been limited to the reports by Sentman et al.^3^, Siefring et al.^7^ and Barta et al.^8^ As such, this study further elaborates our understanding in this field with the multi-instrument measurements covering the different layers of the ionosphere. We discuss below their possible interconnections and associated coupling processes.

### The connection between MCS/thunderstorm, sprites, and GWs

MCS/Thunderstorms are the sources of energy and wave phenomena that can affect the different regions of the earth’s atmosphere. The quasi-electrostatic field generated by strong lightnings within the thunderstorm produces the short-lived sprite events^3,26^, whereas the convective processes within the thunderstorms are the strongest source of GWs^27–29^. Thunderstorms/lightnings are strong sources of charge, current, and electric field^3,30^. The charges and currents associated with thunderstorms directly connect with the lower ionosphere and can generate sprite and trigger early/fast VLF perturbation events. The electrical effects from the thunderstorm can influence the F-region **E** × **B** plasma drift motion and perturb various regions of the ionosphere^30^ due to coupling of the lower and the upper ionosphere. It is conceivable that these processes may operate simultaneously in a thunderstorm with a varying relative contribution. As per the details available in “ MCS Meteorological conditions and sprite observations section”, these pre-existing conditions such as lightning activity and the existence of MCS prevailed during the whole period of the multi-instrument measurements. Altogether the MCS/thunderstorms associated perturbations in the D-, E- and F-region of the ionosphere are supposed to be implied. We do not have the electric field measurement during this time; hence, their possible effects on the F-region **E × B** drift could not be investigated.

In the context of GWs, the convective plume (moist air parcel) model appears to be their important generation mechanism^28^. Convective systems that overshoot the tropopause excite GWs via the rapid displacement of air parcels from the equilibrium^2^. GWs can induce electron density variabilities in the 80–95 km altitude range through wave-breaking and non-linear effects^31,32^. About 3–15 min before the sprite occurrence, we observed GWs in OH imaging that propagated towards their direction and interspersed nearly orthogonally with another set of GWs, dissipated in the background atmosphere, and turbulent regions were observed in the field-of-view. Further, CG lightning stokes (that were not detected by the GLD360 network) acted as a trigger to produce sprite streamers. Siefring et al.^7^ discussed the pre-existing GWs generated by the convective process in the storm. They have found that the tops of extended lines of sprites to be aligned with the GWs troughs seen in OH imaging and suggested that at the trough of GWs ionization rate can be significantly higher, thus suitable for sprite initiation. We could not study such a feature due to a lack of suitable viewing geometry. Siefring et al.^7^ have discussed in detail the importance of a suitable viewing geometry for observations of sprites and associated GWs.

In the airglow imaging, we noted another set of GWs (λ_H_ ~ 60 ± 3 km,υ ~ 89 ± 5 m/s and τ ~ 11 min) arriving from the sprite occurrence direction during 18:21–18:45 UT. First signatures of these GWs were seen during 18:21–18:27 UT over the north horizon i.e., about 9–14 min after the sprite occurrence. With the estimated propagation characteristics, these GWs have travelled a distance of about 48–75 km in 9–14 min duration. First GWs front (marked “S1” in Fig. 3 at time 18:21:12–18:27:11 UT) was about 82 km away from the sprites site. The field-of-view of the imager at OH height was ~ 200 km. Interestingly these observed wave characteristics are in close agreement with the results by Sentman et al.^3^ who reported simultaneous observations of sprites and circular GWs in OH 720–920 nm imaging over an intense Nebraska thunderstorm on the night of 18 August 1999 having λ_H_ ~ 50 km,υ ~ 85 m/s and τ ~ 10 min. Such close similarity points towards GWs generation due to the sprite-associated thermal energy deposition in the mesosphere. However, the perturbation energy estimation analysis by Sentman et al.^3^ does not support such a possibility. Further, unwrapped (i.e., geographically corrected images, not presented here) showed that their fronts were planar. Circular/concentric fronts are expected for a sprite-like point source.

Latent heat release in convective “overshooting” updrafts can generate GWs. Several investigators have reported GWs due to deep convection/convective overshooting associated with thunderstorms/plumes in the MLT region airglow imaging (e.g., Yue et al.^33^, Xu et al.^34^, Chou et al.^35^, Azeem et al.^36^). We inspected cloud top temperature contours during 17:15–18:45 UT to identify deep convective activity at the tropospheric heights. Some of these contours are presented in Fig. 2d–f wherein overshooting of cloud tops (the signatures of deep convective activity and appearing as red patches) were noted close to the sprite location/two lightnings that occurred at 18:12 UT. GWs fronts (marked by S1, S2 and S3 in Fig. 4) arriving from the sprite location indicate their possible source towards the north-northeast direction wherein these two convective patches were located. According to Vadas and Fritts^37^ and Vadas et al.^38^, a convective body generates a continuous spectrum of GWs with the characteristic period *T*_*C*_ given by T_c_ = T_b_ [(D_H_/D_Z_)^2^ + 1]^1/2^ ,where T_b_ is the Brunt-Vaisäla period ~ 4 min at the MLT heights, D_H_ and D_Z_ represent the full horizontal extent and vertical depth of the convective forcing, respectively. The D_Z_ can be roughly taken near the tropopause altitude (~ 18 km) including overshooting updrafts and D_H_ (symbolizing the size of developing overshooting cloud tops) varied from ~ 30 to 110 km. Using these values T_c_ is estimated ~ 8–25 min. GWs periods obtained in airglow, TEC and VLF measurements are 11, 18 and 12.2 min, respectively, which are in good agreement with the estimated characteristic period of GWs generated by two overshooting cloud tops. We also noted appropriate time lag between the convective/lightning activity and the appearance of GWs in OH airglow measurements and dVTEC fluctuations which can be linked to the arrival time of GWs from its source. Azeem et al.^36^, Chou et al.^35^ and Yue et al.^33^ found that GWs started after 15 min, 15–20 min and ~ 1 h of the convective activity, respectively. Barta et al.^8^ also discussed this propagation delay from a thunderstorm to Es altitude and found this delay varies from ~ 20 min to 2 hours. Thus these GWs possibly originated from either of these two developing overshooting cloud tops, however, the planar nature of their fronts can not be explained.

### Troposphere, lower ionosphere, and upper ionosphere coupling process

With regard to the MCS events, two kinds of atmosphere–ionosphere coupling mechanisms have been reported: (1) by the quasi-electrostatic electric fields from lightning activity in the MCS thunderstorm and (2) by MCS thunderstorm generated GWs. The direct heating by lightning associated EMP and/or quasi-electrostatic fields generate TLEs and also cause conductivity modification in the lower ionosphere (D-region)^3,19^. The VLF navigation signals passing through/close to this region are affected by D-region conductivity change and the effect can be seen as short duration perturbations in the amplitude and phase of VLF wave (early/fast event)^39^. This indicates a possible coupling between thunderstorms in the troposphere and the D-region.

The second mechanism is coupling through the convectively generated GWs, as discussed above, the GWs generated by the convection process within the thunderstorm can propagate to the D-region altitude and can cause density fluctuations due to troposphere-lower ionosphere coupling. The present observations do suggest their important role in the troposphere-lower ionosphere coupling.

It is also important to discuss possible coupling between the lower and upper ionosphere (F-region). There is a possibility that wave energy at the lower ionosphere could leak into the upper ionosphere and excite GWs^40^. Walterscheid et al.^41^ have suggested that the thunderstorm-generated GWs can also propagate through to the upper ionosphere supported by the thermospheric temperature gradient. Maurya et al.^9^ reported simultaneous observations of short-period (< 5 min) wave-like signatures in VLF and GPS TEC data representative of the lower and upper ionosphere, respectively. These wave-like signatures were found to be caused by thunderstorm generated convective processes. Thus, the present observations which are the first from the Indian region show that MCS/thunderstorms in the troposphere can significantly perturb the lower and upper ionosphere via GWs.

## Summary

We report rare simultaneous imaging observations of sprites associated with an intense MCS and GWs that indicate their possible interrelationship^3,7^. At upper mesospheric heights, GWs were first observed to propagate towards the northeast direction and dissipated in the background atmosphere. Next, TLE/sprite event occurred over the northern horizon and another set of GWs were observed to arrive from their occurrence direction. The quasi-electrostatic electric fields associated with thunderstorms is the source of TLEs, while GWs can be generated by the MCS associated convection. Such novel observations of GWs before and after the TLEs invoke further studies to understand the possible role of GWs in defining the sprite morphology and GWs generated by their thermal energy deposition.

Further, we noted ~ 11–14 min period GWs in airglow observations while VLF measurements showed GWs with a period ~ 12.2 min. A similar spectrum of GWs was noted in VTEC measurements with a period ~ 18 min, which suggests that MCS/thunderstorms associated GWs can significantly perturb the atmosphere–ionosphere system supporting the D-, E- and F-region coupling besides the electrical coupling. This is the first such report from the Indian subcontinent that features sprites and GWs imaging observations, and the associated D-, E- and F-region coupling.

## Methods and data

Dr. K. S. Krishnan Geomagnetic Research Laboratory (hereafter KSKGRL), located at Prayagraj (formerly Allahabad, ALD) (25.5° N, 81.9° E, geomagnetic lat. ~ 16.5° N) in India, is a regional center of Indian Institute of Geomagnetism (IIG), Navi Mumbai, which was set up to study the lithosphere-atmosphere–ionosphere system near the crest of Equatorial Ionization Anomaly. Several remote sensing experiments have been installed at Prayagraj to observe the atmosphere–ionosphere system (shown in Fig. 1) and brief details of experiments utilized in this study are as follows:

### TLE observations set-up

The TLEs camera system, which recorded the sprite event on the night of 30 May 2014, uses two low light sensitive charge-coupled device (CCD) camera systems^42^. The first camera is of 16 mm F/1.4 wide-angle, horizontal field of view (FOV) of 26.5° and the vertical FOV: 15°. The second camera consists of a 25 mm F/0.95 narrow-angle: horizontal FOV 7.25°, vertical field of view: 4.8° for high resolution event recording. The camera frame rate is 60–250 frames/sec. The digitized video files are time-stamped using a GPS unit providing 1-pulse-per-second (PPS) signal to ensure high precision time stamping of the data. More about the TLE setup can be found in Chanrionet al.^42^ and about the recording set up at Prayagraj (Allahabad) in Singh et al.^43^*.*

### Airglow imaging observations

Co-located and near-simultaneous observations of OI 557.7 nm and OH broadband (705–929 nm) nightglow emissions, emanating from ~ 97 and ~ 87 km, respectively, were made at Prayagraj, India, during May 2014 using an all-sky imager. This imaging system consists of a Mamiya fisheye lens (having ~ 180° field-of-view), a shutter unit, a 6-filter wheel assembly, and a re-imaging optics to form an airglow image in the CCD plane of the detector. *Parihar and Taori*^44^ have described this imaging system and airglow optical filters in detail. OH broadband and 557.7 nm emission were monitored with an exposure time of 60 s at an interval of ~ 6 min. To approximately account for artifacts due to van Rhijn effect and pixel-to-pixel non-uniformity of the CCD detector, flat-fielding of images was performed. Image processing and spatial calibration of airglow images have been discussed elsewhere^16^. On 30 May 2014, clear-sky prevailed during 1430–1930 UT thereby allowing ~ 5 h of good airglow imaging which is used in the present study. OH broadband images shown in Figs. 3 and 4 were time differenced (TD) images [viz. Image_TD_ = Image_Sequence(i)–_Image_Sequence(i-1)_]^16,33^.

### VLF recording setup

The VLF data used in the current work were also recorded at Prayagraj. The VLF receiver is Stanford University developed Automatic Weather Electromagnetic System for Observation Modeling and Education (AWESOME) VLF receiver^45,46^. We have used one-minute average data for the analysis from the recording done at a much higher resolution. The map location of Prayagraj (ALD) station, JJI (22.2 kHz; geog. lat. 32.05° N, geog. long. 131.51° E) VLF transmitter along with their Transmitter Receiver Great Circle Paths (TRGCPs) are shown in Fig. 1. The TRGCP path length for the JJI-PRJ path is ~ 5000 km.

### GPS TEC receiving setup

We have used GPS data from the International GNSS services (IGS) network station, Lucknow (26.9° N, 81.0° E, IGS Site ID: LCK300IND), India. As shown in Fig. 1, Lucknow station is very close to Prayagraj and is located in the MCS region. The data recorded are available in the Receiver Independent Exchange (RINEX) format. The Slant Total Electron Content (STEC) representing free electrons in a column of the unit cross-section between the satellite and the receiver was estimated and further converted to Vertical Total Electron Content (VTEC)^47,48^ using GPS-TEC analysis application software developed at Boston College^49^.

### Lightning data

The lightning data used were obtained from Vaisala Inc. [https://www.vaisala.com/] ground-based global lightning detection (GLD360) network which uses radiated VLF energy lightning strokes to determine the time, location, discharge polarity and radiated peak current of lightning strikes. GLD360 has a detection efficiency of 70%. The median geo-location accuracy of GLD360 is 5–10 km cloud-to-ground strokes^50^. Figure 1 shows the color coded lightning activity evolution during 16–24 UT using GLD 360 data.

## Supplementary Information

Supplementary Information 1.

Supplementary Video S1.
