# Supplementary material for: Rare observations of sprites and gravity waves supporting D, E, F-regions ionospheric coupling
Source: Sci Rep. 2022 Jan 12;12:581. doi: 10.1038/s41598-021-03808-5 (PMC8755841; doi:10.1038/s41598-021-03808-5)
Supplement: Supplementary file 1 — Supplementary Information 1. [file 41598_2021_3808_MOESM1_ESM.pdf]

Rare observations of Sprites and Gravity Waves supporting D, E, F-regions ionospheric coupling

Ajeet K Maurya[1],Navin Parihar[2], Adarsh Dube[3],Rajesh Singh[3*], Sushil Kumar[4], Olivier Chanrion[5], Maja Tomicic[5], Torsten Neubert[5]
[1]Department of Physics, Doon University, Dehradun, India
[2]Equatorial Geophysical Research Laboratory, IIG, Tirunelveli, Tamilnadu, India
[3]KSK Geomagnetic Research Laboratory, IIG, Prayagraj (Allahabad), India
[4]The University of South Pacific, Suva, Fiji
[5]National Space Institute, Technical University of Denmark (DTU Space), Elektrovej 327, 2800 Kgs. Lyngby, Denmark
*Correspondence to rajeshsing03@gmail.com

Caption to Supplementary Materials:

(1) **Supplementary Movie S1:** Movie created from TD OH broadband images during 14:52-19:03 UT showing gravity wave activity before and after the TLE/MCS event.

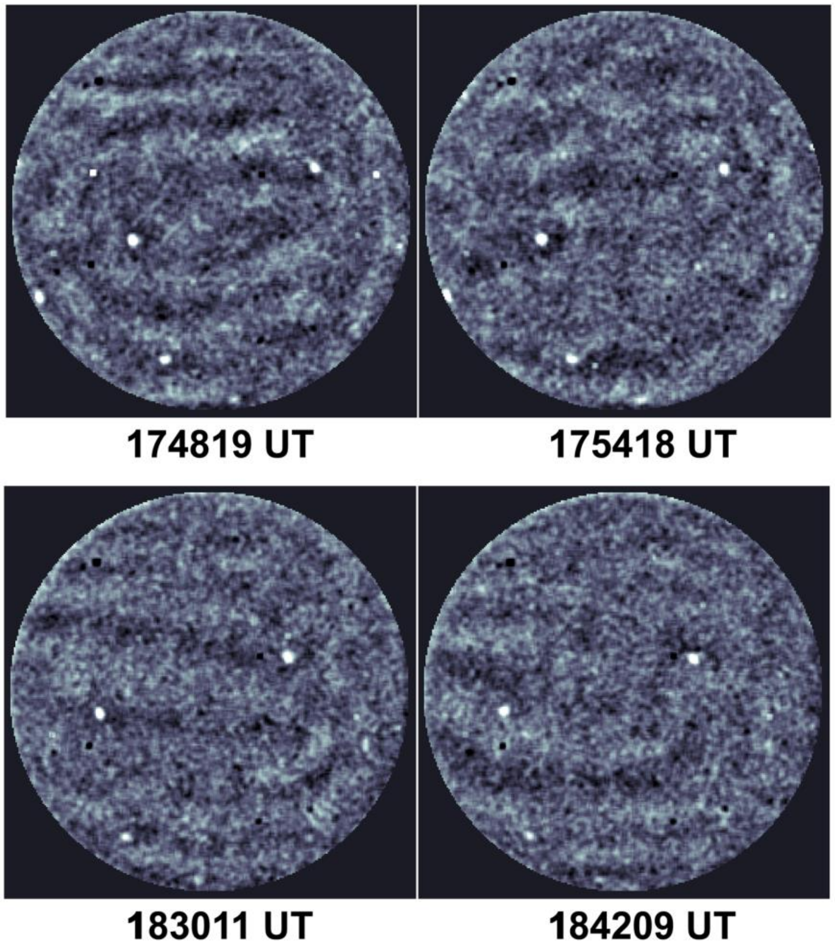

(2) Supplementary Figure S2: Selected TD OI 557.7 nm images at 17:48-17:54 UT and 18:30-18:42 UT showing faint signatures of gravity wave activity before and after the TLE/MCS event.
